# Epigenetics in Neurological and Psychiatric Disorders: A Comprehensive Review of Current Understanding and Future Perspectives

**DOI:** 10.7759/cureus.43960

**Published:** 2023-08-23

**Authors:** Han Grezenko, Chukwuyem Ekhator, Nkechi U Nwabugwu, Harshita Ganga, Maryam Affaf, Ali M Abdelaziz, Abdur Rehman, Abdullah Shehryar, Fatima A Abbasi, Sophia B Bellegarde, Abdul Saboor Khaliq

**Affiliations:** 1 Translational Neuroscience, Barrow Neurological Institute, Phoenix, USA; 2 Neuro-Oncology, New York Institute of Technology, College of Osteopathic Medicine, Old Westbury, USA; 3 Public Health, Hudson College of Public Health, University of Oklahoma Health Sciences Center, Oklahoma City, USA; 4 Neurology, William Mason High School, Mason, USA; 5 Internal Medicine, Women Medical College, Abbottabad, PAK; 6 Internal Medicine, Alexandria University Faculty of Medicine, Alexandria, EGY; 7 Surgery, Mayo Hospital, Lahore, PAK; 8 Internal Medicine, Allama Iqbal Medical College, Lahore, PAK; 9 Cardiology, Shifa International Hospital Islamabad, Islamabad, PAK; 10 Pathology and Laboratory Medicine, American University of Antigua, St. John's, ATG

**Keywords:** neurology, future educational reform, future prospect, public health care, public health, clinical psychiatry, human genetics and epigenetics

## Abstract

The burgeoning field of epigenetics offers transformative insights into the complex landscape of neurological and psychiatric disorders. By unraveling the intricate interplay between genetic, epigenetic, environmental, and lifestyle factors, this comprehensive review highlights the multifaceted nature of mental health. The exploration reveals the potential of epigenetic modifications to revolutionize our understanding, diagnosis, treatment, and prevention of these disorders. Emphasizing the importance of multidisciplinary collaborations, large-scale studies, technological advancements, and ethical considerations, the review asserts the promise of epigenetics as a vital tool for personalized medicine, early intervention, and public health strategies. While acknowledging the challenges in a still-emerging field, the review paints an optimistic picture of epigenetics as a groundbreaking approach that can reshape mental healthcare, offering hope for those affected by neurological and psychiatric conditions. The future trajectory of the field relies on interdisciplinary efforts, ethical diligence, innovative technologies, and translating scientific insights into real-world applications, thereby unlocking the vast potential of epigenetics in mental health.

## Introduction and background

Epigenetics refers to changes in gene function that do not alter the underlying DNA sequence, and these changes can be heritable as well as often reversible. Such modifications include processes like DNA methylation, histone modification, and non-coding RNA regulation [1]. Far from being limited to normal development and cellular differentiation, epigenetic processes have been linked to a broad range of diseases such as cancer and cardiovascular diseases. More recently, they have been connected to neurological and psychiatric disorders [1,2]. This complex interaction between genes and the environment has illuminated the pathogenesis of neurological and psychiatric disorders through epigenetic mechanisms. Unlike conventional genetic studies, which have frequently fallen short, epigenetic research opens new pathways to understanding the co-occurrence and comorbidity of these conditions [3].

The current review harbors specific aims, including the exploration of the existing knowledge concerning the role of epigenetics in individual neurological and psychiatric disorders. This examination emphasizes both the commonalities and differences within these fields. Additionally, this review intends to delve into the shared epigenetic pathways and mechanisms that intersect these disorders, offering insights into the current challenges and future directions in studying epigenetics in co-occurring neurological and psychiatric conditions. Covering key disorders such as Alzheimer's disease, Parkinson's disease, depression, and schizophrenia, the scope of this review extends to the broader implications for understanding and treating co-occurring disorders.

A compelling rationale motivates this review. The evidence continues to grow that neurological and psychiatric disorders are not merely distinct entities but often co-occur, bound by common underlying epigenetic mechanisms [4]. Grasping this intricate interconnection is vital to advance diagnosis, treatment, and prevention strategies. Through an all-encompassing overview of the existing literature, pinpointing the existing gaps and challenges, this review seeks to enhance an integrated and personalized approach to treating these multifaceted and complex disorders.

## Review

A brief overview of epigenetics

Epigenetics, defined as the study of changes in gene function that don't alter the underlying DNA sequence but significantly influence gene expression, encompasses several key mechanisms [5]. One of these is DNA methylation, which involves the addition of a methyl group to the DNA's cytosine base and often leads to the silencing of the gene [5]. Another mechanism is histone modification, which refers to alterations in the histones, the proteins around which DNA is wound. Such changes affect the accessibility of genes for transcription and are central to gene expression regulation [6]. Additionally, the regulation of small non-coding RNAs plays a vital role in controlling gene expression after the transcription process [7]. These dynamic mechanisms are crucial in various biological contexts, including development, differentiation, tissue specificity, and responses to environmental changes. Importantly, they can be influenced by factors such as environmental exposure, aging, and disease states [5,6].

The implications of these epigenetic mechanisms are particularly profound in the fields of neurology and psychiatry. In neurological disorders, including Alzheimer's, Parkinson's, and Huntington's diseases, epigenetic changes are integral to disease onset, and progression, and may even serve as targets for therapeutic intervention [8,9]. Similarly, in psychiatric conditions such as depression, schizophrenia, and bipolar disorder, epigenetic alterations are believed to play a significant role in disease susceptibility, especially in the context of the interplay between genetic and environmental risk factors [10,11]. This epigenetic understanding is not limited to isolated conditions; there is emerging evidence suggesting that shared epigenetic pathways may explain the co-occurrence of neurological and psychiatric conditions. Such insights might pave the way for more integrated diagnostic and treatment approaches, thereby revolutionizing the care for these complex disorders [4,12].

The study of epigenetics offers novel insights into disease etiology, progression, and potential therapies in both neurological and psychiatric contexts. By recognizing the individual's unique epigenetic landscape, epigenetic research opens the door for the development of personalized medicine approaches that hold immense promise for enhancing patient care [13].

Co-occurrence of neurological and psychiatric disorders

Epidemiology

Understanding the epidemiology of co-occurring neurological and psychiatric disorders directly impacts patient care. Recognizing these connections allows for earlier detection of symptoms, leading to accurate treatment planning and improved quality of life. As our understanding of these relationships grows, so does our ability to provide comprehensive care that acknowledges human complexity [14,15].

The co-occurrence of neurological and psychiatric disorders is increasingly recognized. Neurological conditions often come with psychiatric symptoms. For instance, about 40% of Parkinson's disease patients experience depression, significantly affecting their life quality [16]. Similarly, nearly half of Alzheimer's disease patients often suffer from anxiety and depression [17].

Other notable instances of this co-occurrence include epilepsy, where psychiatric disorders such as depression and anxiety are prevalent in about 30% of individuals [18]. Furthermore, traumatic brain injury (TBI) often leads to psychiatric conditions, including post-traumatic stress disorder (PTSD) [19]. These examples illustrate the significant interplay between neurological and psychiatric conditions.

Biological Links

The intricate biological connections between neurological and psychiatric disorders encompass a wide array of factors, including genetics, neurochemistry, epigenetics, and even alterations in brain structure and function. These links not only help explain the co-occurrence of these disorders but also emphasize the necessity for a holistic approach to their diagnosis and treatment.

Shared genetic factors contribute to the co-occurrence of neurological and psychiatric disorders due to common genetic pathways [4]. Dysregulation of neurotransmitter systems like serotonin and dopamine is another link, potentially causing symptoms in both disorder types [20]. Shared epigenetic mechanisms add complexity, influencing disorder susceptibility and progression [12]. Changes in brain structure and function, like specific region atrophy in Alzheimer's disease, can contribute to cognitive decline and psychiatric symptoms like depression [21].

The complex biological links between neurological and psychiatric disorders call for an integrated approach, leading to more effective, personalized therapies. Ongoing research and increased understanding of these links offer hope for future care advancements, emphasizing the potential for a nuanced approach to managing these often co-occurring, interconnected disorders [22].

Epigenetics in specific disorders

Neurological Disorders

Epigenetics is key in understanding many neurological disorders, providing insights into their mechanisms and potential therapies. In Alzheimer's disease, specific epigenetic changes like DNA methylation and histone modifications contribute to disease progression [9]. In Parkinson's disease, DNA methylation changes are associated with both familial and sporadic forms, highlighting the broad impact of epigenetic factors [9].

Huntington's disease, a devastating genetic disorder, has also been linked to epigenetic changes, particularly abnormal histone modifications. These alterations are thought to be part of the complex underlying mechanisms that lead to the symptoms and progression of the disease [23]. Even in multiple sclerosis, a chronic inflammatory disorder affecting the central nervous system, epigenetic factors are believed to play a role, contributing to its development and progression through intricate gene-environment interactions [24].

Psychiatry Disorder

Epigenetics is crucial in understanding and treating psychiatric disorders, revealing the interplay between genetics, epigenetics, and environmental factors. Epigenetic changes in genes related to neurotransmitter systems and stress response have been found in depression, offering new treatment approaches [25]. In schizophrenia, DNA methylation in genes involved in neural development and neurotransmission has been observed [26]. Epigenetic marker alterations have been identified in bipolar disorder, providing insights into its complex biology [27]. In autism spectrum disorders (ASD), aberrant DNA methylation patterns have been implicated [28]. These findings highlight the complex nature of psychiatric disorders, where genetic, epigenetic, and environmental factors interact. Unraveling these connections advances scientific knowledge and paves the way for targeted therapeutic interventions. Recognizing epigenetics' role in psychiatric disorders promises future research and treatment, emphasizing the need for a comprehensive approach considering these conditions' complexity.

Co-occurrence

The co-occurrence of neurological and psychiatric disorders is complex, and epigenetics may offer insights into why these disorders often coexist and how they could be more effectively treated. Certain epigenetic changes may be common to both disorder types, contributing to their co-occurrence [29]. These shared epigenetic markers could bridge the gap between the two disorder types in traditional diagnostic frameworks. Cross-disorder analysis of epigenetic modifications may help identify overlapping pathways and mechanisms [30]. This broader epigenetic landscape exploration could uncover the biological basis of co-occurrence. Understanding the shared epigenetic landscape could lead to novel therapeutic approaches that target both symptom types simultaneously, providing more holistic and integrated care for individuals with these often overlapping conditions [31].

Impact of environmental factors

The understanding of both neurological and psychiatric disorders has deepened significantly with the recognition of the intricate interactions between genetic, epigenetic, and environmental factors. A notable aspect of this complex interplay lies in the impact of various environmental factors on the epigenetic changes associated with these disorders, particularly in the realm of lifestyle choices.

Lifestyle Factors

Lifestyle factors like diet, exercise, substance abuse, and stress significantly influence the epigenetic landscape, impacting neurological and psychiatric conditions. Nutritional imbalances can alter DNA methylation patterns, contributing to disorders like depression and Alzheimer's disease [32]. Regular physical activity modifies epigenetic markers in genes related to brain health and cognitive function [33]. Substance abuse can induce epigenetic changes affecting neural pathways, contributing to addiction and co-morbid psychiatric disorders like depression and anxiety [34]. Chronic stress has been linked to epigenetic modifications in genes regulating the stress response, leading to conditions like anxiety and depression [35]. These findings highlight the intricate biological pathways through which our experiences shape our mental health.

Early-Life Exposure

Environmental factors influence neurological and psychiatric conditions from early life stages. Early-life exposures, including prenatal exposures, childhood trauma, and environmental toxins, can induce long-lasting epigenetic effects, potentially leading to disorders later in life. Prenatal exposures like maternal stress, malnutrition, and toxin exposure can induce epigenetic changes affecting neurodevelopment and risk for disorders like autism and schizophrenia [36,37]. This highlights the importance of prenatal care for long-term mental health.

Childhood trauma can cause long-lasting epigenetic alterations, increasing the risk for depression, anxiety, and PTSD [38]. This underscores the need for trauma-informed care and interventions addressing these epigenetic changes. Exposure to environmental toxins like lead and air pollution during critical developmental periods can alter the epigenome, contributing to neurodevelopmental and cognitive disorders [39]. This connection between environmental quality and mental health calls for societal engagement to reduce pollution and environmental toxins for mental and neurological health.

Clinical implications

Understanding the role of epigenetics in neurological and psychiatric disorders provides valuable insights with wide-ranging clinical implications. Here, we will explore these implications in terms of diagnosis, treatment, and prevention.

Diagnosis

Epigenetics' application in diagnostics offers a new frontier in understanding and treating neurological and psychiatric conditions. Epigenetic modifications, like changes in DNA methylation patterns, can serve as biomarkers for various disorders, enabling earlier and more accurate diagnoses.

Biomarker identification, where unique epigenetic modifications can be detected before symptom onset, is one area where epigenetics is impactful. For instance, specific DNA methylation patterns have been proposed as diagnostic tools for Alzheimer's disease [40]. Early detection allows for interventions at earlier stages, potentially slowing or preventing symptom onset [41].

Epigenetics also enables a more personalized approach. Epigenetic profiling provides a snapshot of an individual's unique interplay of genetic and environmental factors, allowing for a nuanced understanding of disease risk and progression [42]. This personalized diagnosis recognizes diseases as influenced by a complex and unique combination of factors in each individual, leading to more targeted and effective treatments.

Treatment

Epigenetics is transforming the treatment landscape for neurological and psychiatric disorders, offering a platform for developing new, targeted, and individualized therapies. This transformative potential is illustrated in targeted therapies, personalized treatment plans, and combination therapies. Targeted therapies use the reversible nature of epigenetic changes, aiming interventions at specific modifications responsible for disease symptoms. This precision medicine approach has shown promise in treating conditions like depression and schizophrenia, with interventions targeting particular epigenetic alterations [29,43].

Personalized treatment plans use precision medicine to create therapy regimens tailored to each patient. Understanding an individual's unique epigenetic makeup allows clinicians to devise treatment strategies optimized for that patient's specific situation, enhancing efficacy and reducing potential side effects [44].

Combination therapies integrate epigenetic therapies with traditional pharmacological and behavioral interventions, creating a more comprehensive treatment plan that addresses the disease on multiple levels [45]. For example, epigenetic therapy might correct a specific molecular pathway, while behavioral therapy could address lifestyle factors contributing to the disease. This multifaceted approach recognizes the complexity of neurological and psychiatric disorders, requiring a multifaceted treatment strategy.

Prevention

Epigenetics is opening new horizons in the prevention of neurological and psychiatric disorders, shifting focus towards prevention rather than merely treating symptoms. This preventative paradigm is evident in early intervention, lifestyle modification, and policy and public health strategies.

Early intervention involves identifying epigenetic markers linked to disease susceptibility, enabling measures to minimize risk or delay disorder onset, especially in genetically predisposed individuals [46]. This approach aims to recognize and respond to a disease process at its earliest stages, potentially improving outcomes and averting full disorder manifestation.

Lifestyle modification, based on the understanding that lifestyle factors like diet, exercise, and stress influence epigenetic changes, is another key prevention area [47]. Implementing targeted behavioral and nutritional interventions promoting healthy epigenetic patterns may protect individuals against developing these complex disorders. This approach empowers individuals to control their health, using epigenetic insights to make informed lifestyle decisions that may mitigate disease risk.

Policy and public health strategies incorporating epigenetic principles can form a broader societal approach to prevention [48]. Understanding how environmental factors like toxins and community dynamics shape the epigenome can inform public health policy creation and implementation. For example, regulations could be instituted on toxins known to cause harmful epigenetic changes or community-based mental health programs could be developed to address broader social health determinants. This approach recognizes prevention as a societal responsibility, requiring coordinated policies and interventions addressing these disorders' root causes on a community-wide level.

Future perspectives

The integration of epigenetics into the understanding of neurological and psychiatric disorders opens new horizons for both research and clinical practice. Here, we highlight the future perspectives, focusing on the research needs and potential clinical applications.

In the burgeoning field of epigenetics, particularly as it pertains to neurological and psychiatric disorders, there are several vital areas where research needs to be intensified. The following areas represent key priorities for the continued advancement of our understanding and our ability to intervene effectively in these complex conditions.

Firstly, future research must actively embrace multidisciplinary collaborations. Integrating knowledge and methodologies from genetics, epigenetics, neuroscience, psychology, and environmental sciences will be essential to develop a comprehensive understanding of these multifaceted disorders. The complexity of neurological and psychiatric conditions demands an equally complex and nuanced research approach, one that breaks down traditional disciplinary silos and fosters innovation through interdisciplinary synergy [49].

Large-scale studies represent another crucial research need. To detect subtle epigenetic changes that might only manifest in a small subset of the population and to validate potential biomarkers, research must be conducted on a scale that allows for robust statistical analysis. These large-scale population studies will provide the statistical power necessary to unearth the nuanced relationships between epigenetics and disease [50].

Longitudinal investigations, tracking epigenetic changes over time, particularly from prenatal stages through adulthood, will provide vital insights into how these modifications contribute to disease onset and progression. This temporal perspective offers a unique vantage point to observe how epigenetic changes evolve and interact with other factors over time, revealing the dynamic nature of these processes [51].

Technological advancements in epigenetic analysis are also an area in need of continued development. Tools such as next-generation sequencing have revolutionized our ability to analyze the epigenome, but further innovations will enable even more precise and extensive investigations. These advancements will push the boundaries of what is currently possible, facilitating research that is more nuanced, more detailed, and more impactful [52].

Lastly, ethical considerations must be at the forefront of all research efforts. The handling of sensitive genetic and epigenetic information raises profound ethical questions, particularly around issues of privacy and consent. Strong ethical guidelines must be in place to govern this research, ensuring that the pursuit of scientific understanding does not come at the expense of individual rights and societal values [53].

Clinical application

The emerging insights from epigenetics have begun to reshape our understanding of neurological and psychiatric disorders, offering new pathways for clinical applications. These applications extend beyond research laboratories, finding tangible and impactful roles in the clinical setting.

First and foremost, the potential for early detection and intervention through epigenetic markers holds immense promise. By identifying individuals at risk based on their unique epigenetic signatures, clinicians can initiate timely interventions. This may range from preventative measures to early-stage treatments that can either prevent the onset or mitigate the severity of neurological and psychiatric conditions. This proactive approach could be a significant step forward in shifting from reactive to preventive healthcare, fundamentally altering how these disorders are managed [54].

The arena of personalized medicine is also being revolutionized by epigenetics. By incorporating epigenetic profiling into clinical practice, healthcare providers can devise therapeutic strategies that are specifically tailored to an individual's unique genetic and epigenetic makeup. This approach maximizes treatment effectiveness while minimizing potential adverse effects, moving away from a one-size-fits-all model to a more nuanced, patient-centered care paradigm [55].

Epigenetics also has broad implications for community and public health programs. The knowledge gleaned from epigenetic research can guide community-based interventions and inform public health policies. These efforts can be targeted to address the unique needs and challenges of different social and economic settings, promoting mental well-being on a societal level. Whether it's through informed regulation of environmental toxins or community mental health initiatives, the insights from epigenetics offer a powerful tool for public health strategists [56].

Finally, the role of education and awareness cannot be overlooked. Educating healthcare providers, patients, and the general public about the role of epigenetics in mental health fosters a more informed healthcare environment. With a deeper understanding of how lifestyle, environment, and genetics intertwine, individuals can make more proactive and informed healthcare decisions. Education is the bridge that connects scientific discovery to everyday healthcare, and in the realm of epigenetics, it's a bridge that can lead to more enlightened and effective care [57].

## Conclusions

The exploration of epigenetics in the context of neurological and psychiatric disorders has unveiled a new dimension of understanding, offering a more comprehensive perspective on the complexity and adaptability of the human brain. This review has highlighted the pivotal role of epigenetic modifications in shaping individual vulnerability and resilience to these disorders, underscoring their significance in diagnosis, treatment, and prevention strategies. The potential of epigenetic markers to revolutionize diagnostic processes, coupled with the promise of epigenetically targeted therapies, opens up new avenues for more personalized and effective healthcare.

Furthermore, insights into the influence of lifestyle and environmental factors on the epigenome provide valuable clues for devising preventative measures and crafting informed public health policies. Despite the significant progress made, the field of epigenetics remains in its infancy, teeming with unexplored avenues and challenges. The realization of its potential hinges on continued interdisciplinary collaboration, technological innovation, adherence to ethical principles, and the practical application of scientific discoveries. In conclusion, the transformative potential of epigenetics offers a beacon of hope for those burdened by neurological and psychiatric disorders, underscoring the value of this research as a precious addition to the existing literature.
